# Clinical Notes on Intemperance and Its Prevention

**Published:** 1862-08

**Authors:** Robert Druitt

**Affiliations:** M. R. C. S., London


### CLINICAL NOTES ON INTEMPERANCE AND ITS PREVENTION.

By ROBERT DRUITT, M.R.C.S., London.

I have hitherto spoken of the bodily sensations which lead to intemperance; but the mental are of infinitely greater consequence. And here let me notice the most cruel and unreasonable neglect with which minor mental affections are treated. I speak not of what is formally called "insanity,' but of the nervous wretchedness, the bad spirits, the dejection and groundless fears of the feeble and hypochondriacal. If a woman have a pain in the side, or a spot on the skin, her husband will immediately seek medical relief for her, though her bodily sufferings be but slight, and her mental none at all. She may be essentially as cheerful as ever, spite of a little pain, which she readily bears. But let the same woman be ill in that which constitutes her very inner self; let her thoughts be gloomy, hopeless, and self-tormenting, so that her life is as miserable as if she were a prey to remorse for the most grievous crimes, and then what is done for her? Either she is despised as a fool, and told so, or she is argued with; just as if any one would entertain irrational fears if they were capable of being argued away. Or she is scolded for sinful despondency, and told that she is wicked to despair. Or she is told to drink; to have a glass of port wine when she feels low—or all these things may happen to her in turn, till, at last, the husband coming home unexpectedly some day, finds his wife drunk! Christian friends then wonder how a lady could so degrade herself; all the blame is laid on the victim's head, and not one word said about those who have so cruelly neglected her, through utter ignorance of mental physiology, and blindness to the things going on under their eyes.

A man lately brought his wife to me, saying, "Really, sir, I don't know if it is of any use to ask you to see her; there is absolutely nothing the matter with her, but she is such a fool. She is moping and crying all day, with nothing on earth to cry about. She has every comfort that a woman can have, and yet is never happy, though I keep telling her how wicked and foolish she is."

I once saw a lady who had been found out in a drunken fit. Her relatives were sent for. Her mother and aunt came; the former thrashed her with her fists; the latter pounded her with texts of Scripture, such as, "The wicked shall be turned into hell," and about "a lake of fire reserved for liars and drunkards," etc., and this to a poor wretch, who fled to the gin bottle for relief from inward misery that was uncared for by those about her, and so timid, as she told me, that she had scarcely courage to give an order to her own maid. From what I have seen of the secret solitary drinkers, it is vain to attempt to terrify them with notions of punishment, present or future; there is no state that they can conceive of more wretched than that which exists within them, and for which they seek a short respite in drunkenness.

Mental suffering is far worse to bear than bodily, and it is almost as common, especially amongst the educated classes. It is really the source of that disposition on the part of educated and refined persons to encourage quacks, of which regular practitioners complain. When regular practitioners cease to utter the formula, "Madam, I assure you your complaints are purely nervous," and with that formula to dismiss the case as unworthy of notice, then they will have a right to complain that their patient seeks from a quack the solace which they will not stoop to administer.

Of the forms of mental suffering, short of what is called "insanity," yet frightfully common, more distressing than any bodily illness, more incurable, and most pernicious—in tempting women to drink, I have already mentioned *nervousness*. The best description of it was given by Charles Lamb. "Business," he says, "which I once used to enter upon with some degree of alacrity, now wearies, affrights, and perplexes me. I fancy all sorts of discouragements, and am ready to give up my occupation which gives me bread, from a harassing conceit of incapacity. The slightest commission given me by a friend, or any small duty which I have to perform for myself, as giving orders to a tradesman, haunts me as a labor impossible to be got through." To the mental are added bodily symptoms of the same sort, as the shaking hand and unsteady sight. These symptoms may be caused by bodily weakness alone, by worry of mind, by use of narcotics, and more especially of alcohol, by green tea, and equally by too much sleep. The hours during which they are most tormenting, are between early morning and the mid-day meal. The difficulty in relieving them arises from the difficulty of coercing a feeble stomach, reeking with the dregs of yesterday's mal-assimilation, to receive and digest food early in the day. Hence, wine and spirits are fatally tempting. Of this again, when I speak of treatment.

Whoever lives without risk of suicide, has passing through his brain during waking hours an easily flowing succession of thoughts, the result of which is a comfortable degree of self-reliance, self-satisfaction, and self-respect. But illness, anxiety of mind, and self-reproach at misfortunes unavoidable or not, may enfeeble the brain, and produce a train of thought which is positively painful, and requires effort to bear patiently. They next enfeeble those powers by which we distinguish between matter of fact and matter of imagination. There is many a case of ill-nourished brain seen in daily practice, between which and melancholia the line of demarcation is not well marked.

In May, 1854, a medical friend called on me, bringing with him the last volume of the Medico-Chirurgical Transactions, which he opened, and asked me to look at a paper by Dr. S. Merriman "On Inversion of the Womb." He looked as wretched as possible, and told me that a very important patient of his, whom he had recently attended, and whose good word was of the highest consequence, had met with this accident, and that he was afraid to reveal it, but that out it must come soon, and that the patient would die and himself be ruined. Of course I condoled with him, and asked particulars. But the odd thing was that I could get no particulars; the inversion had been taken for granted. So I advised my friend, instead of going and confessing to the husband, to take an opportunity of examining the patient, to find whether the womb were really inverted or no. He did so, and found that he had been disquieting himself about a mere fiction of the imagination. He had been suffering from loss of rest, irregular meals, and great anxiety of mind, and had got his brain into so feeble a state that it was enslaved by an hallucination. Rest, good living, and wine speedily cured him; but it is a singular fact, that two years later he got the same crotchet into his head respecting another patient about whom he had been much harassed.

This case may be an example of the morbid fancies which haunt the brain of the wretched hypochondriac, unable to weigh and dismiss them with a clear judgment. The commonest is the notion that other persons' words and actions are intentionally offensive. In such cases, whether the evil be great or little, alcoholic liquids strengthen the judgment, change the current of thought and render it agreeable, beget a comfortable sense of self-respect, and enable the patient to bear his woes. Hence, the use of them may be, first, a dangerous remedy; next, an inveterate aggravation of the original malady.

The process by which *grief* leads to drinking is short and easy as the path to Avernus. There is no doubt but that grief—the condition caused by the news of some great calamity—produces effects on the brain of a physical character. The sufferer feels a sudden rush of blood to the head; a faintness; a dull pain; a sense as if the head would burst and the eyes start from their sockets; a stunned feeling, followed by a soreness; and inability to control the thoughts, which roll round and round in one distressing circle, without the power to dismiss them and entertain others. Between a man who receives shocking news and one who receives a blow from a stick, there is this difference, the physical injury is over at once; the mental persists and may grow in virulence. To the mental torture is soon superadded bodily illness; and the effects of grief which I have seen have been in one of these forms.

There may be, first, the effects of exhaustion—of the great wear and tear of the brain. This was well described to me some time ago by a widow. Intense occupation in nursing a sick husband had ceased suddenly with his death. "When the house is shut at night," she said, "and everything quiet around me, then my sad thoughts come pouring down upon me, and go on, round and round, and over and over again. After a time, as I lie tossing about, a most horrible faint sinking comes over me, as if my heart and inside were clean gone, and I was sinking into the earth, bed and all. Then I must have brandy. I get out and take it, and sleep and feel better; if I do not, I am good for nothing the next day."

The second effect of grief is shown in those disorders of the blood which seem to result from admixture with it of organic matter, the result of nervous wear and tear. The usual symptom is sick headache; stunning headache, with vomiting of bile, or of acid stuff, or both, and large elimination of uric acid by the kidneys. Boils notoriously may be induced by grief; so may gout. I once was told by a man that a fit of vexation from disappointed love had settled in his nose and produced carbuncle there. It was quite true. There is much reason, too, for believing that grief may conduce to cancer.

Thirdly, there are more or less permanent disorders of the nervous system. There is a form of hysteria in which pains are, as vulgarly said, "similated." I recollect seeing a young lady bathed in tears, and complaining of violent pain in the side. She expanded her chest in such a way as to show that the cause of pain was not seated in the side; but the mind was deeply wounded; she was ill; sympathy was a necessity to her; the pain was but a reflex effect of a broken heart. This, as I humbly conceive, is the key to the obstinate, inscrutable, and apparently utterly abnormal pains and local complaints of hysteric young women. As a sailor will feel pain in the legs which were cut off years ago, as disease of the nervous centres may cause pain in the extreme nervous branches, so a mind deeply wounded, especially with a grief that must be hidden, produces some outward local pain.

Hence the obstinate pain in the knee, or ankle, or spine, the spasms, and other symptoms of severe hysteria. I have seen cases, from long-hidden grief, of utter fatal atrophy of body and imbecility of mind; such patients might have been saved by a greater use of wine, under medical direction; but yet might probably have become confirmed intemperates. Many a man lives as an intemperate, who, but for alcohol, would have died. In counting up the evils of alcohol, it is but fair to substract from them those which it finds and alleviates.

Grief fills the mind with images which are positively noisome. *Stagnation*, in which the mind is simply not supplied by any images at all, is as bad or worse. Such is the condition of persons of middle age who have lost hope; whose schemes of life are blighted; who have failed in their calling, and are too old to choose another. Such are the physicians at sixty who hate the profession, and wish they had gone to the bar; such the barristers who "always felt they had a taste for physic;" and the soldiers who say that the service is clearly going to the devil, and that only incompetent fools get promotion. Such are, too, many of the inmates of almshouses, refuges, and colleges for decayed professional men or their widows. Such are many of the single women of limited income who live in those protestant convents, the boarding-houses that abound in the suburbs of London. Such are the shepherds in the Australian bush, who, after months of monotony, must go and get drunk, to save their lives by a little excitement.

Of the effects of anxiety of mind, the following may be an example. A patient writes: "I received intelligence which caused me intense anxiety, which lasted several days. Absorbed though I was, I could not help noticing my bodily sensations. After a fit of more anxious musing than usual, I had such singing in my ears, giddiness, etc., feeling as if all my flesh was asleep. My feet felt partly as if they had pins and needles, and partly as if there were wool inside the soles of feet. And such a craving for food and drink!"

But there are certain perturbed states of the nervous system which directly lead to drinking, and which seem not to be engendered by outward circumstances, but by some congenital defect in the original formation or in the development of the nervous system. Such are the cases which may be called "primary hysteria," and (with certain reservations) "moral insanity." There are young persons of either sex, whose nervous system is in such state that the ordinary occupations and surroundings which give happiness to others, give none to them. They are wretched and dissatisfied, and seek for some additional means of excitement. That excitement is found in two modes—in tormenting others, and indulging themselves. There are children who from tender age do the most malicious and mischievous acts, merely to gratify themselves. Such was a young lady who, when on a visit, hid the dressing-case of another visitor; such are the girls who set fire to houses; and others, of whom a good example may be cited—the unfortunate and notorious girl to whom the name of the "Female-Jesuit" was ignorantly and slanderously given, and whose history was published with ludicrous simplicity by a miserable preacher upon whom she had quartered herself, and whom she deluded and tormented for months. Not long ago, I was consulted about a young lady, rich and good-looking, but restless, always craving for excitement; able to have almost every indulgence that money could procure, and to travel wherever she liked, yet secretly a drinker.

Here I must stop for the present. Let me sum up by saying, that as of bodily ailments, so of the minor mental perturbations met with in common life, there is a vast amount whose natural tendency is to seduce into intemperate habits, because alcoholic liquors give temporary relief. Such mental ailments are too often treated by scolding, argument, or *le prêche.* They ought to receive the most refined and careful medical attention; and if they did, many a case of suicide, insanity and drunkenness would be prevented.—*Med. Times and Gazette.*

